# Supplementary material for: Influence of Chitosan, Salicylic Acid and Jasmonic Acid on Phenylpropanoid Accumulation in Germinated Buckwheat (Fagopyrum esculentum Moench)
Source: Foods. 2019 May 6;8(5):153. doi: 10.3390/foods8050153 (PMC6560396; doi:10.3390/foods8050153)
Supplement: Supplementary file 1 [file foods-08-00153-s001.pdf]

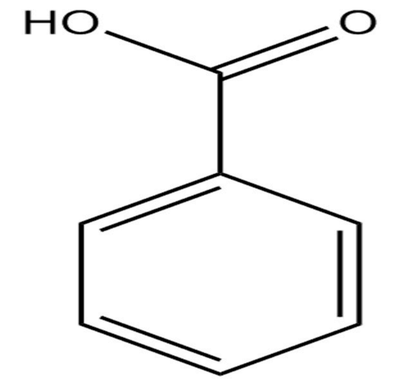	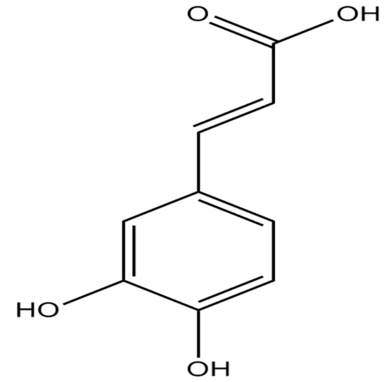	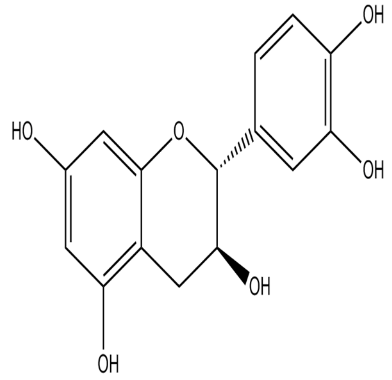
Benzoic acid	Caffeic acid	Catechin
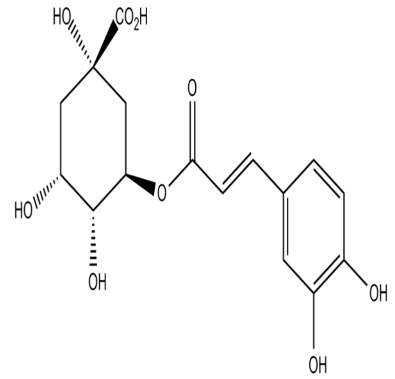	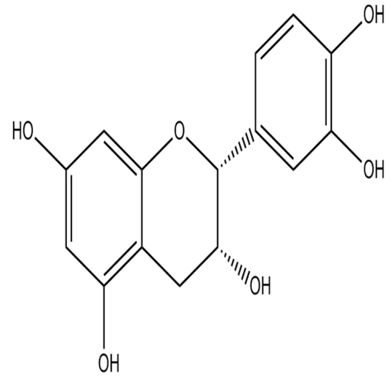	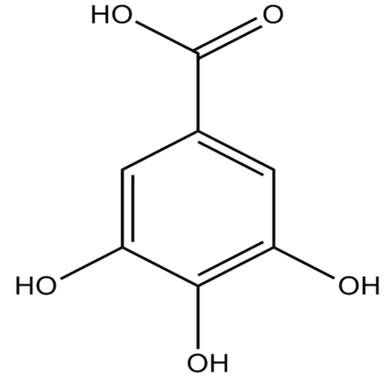
Chlorogenic acid	Epicatechin	Gallic acid
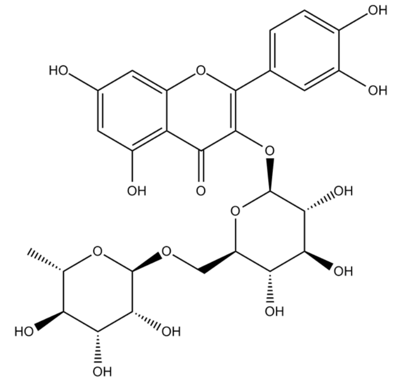		
Rutin		

Figure S1. Chemical structures of the phenolic compounds and benzoic acid.

Table S1. The effect of 0.1% chitosan and 150 μM jasmonic acid on the growth of germinated b uckwheats grown for 72 hours.

	Fresh Weight (g)	Dry Weight (g)
Control	8.98 ± 0.63 a[1]	3.29 ± 0.16 a
0.1% chitosan	8.37 ± 0.22 a	3.08 ± 0.04 a
150 μM jasmonic acid	8.63 ± 0.37 a	3.18 ± 0.11 a

[1] Means with different letters in the same column differ significantly ($p < 0.05$, ANOVA, Duncan Multiple Range Test (DMRT).
